# Human Migration Patterns in Yemen and Implications for Reconstructing Prehistoric Population Movements

**DOI:** 10.1371/journal.pone.0095712

**Published:** 2014-04-23

**Authors:** Aida T. Miró-Herrans, Ali Al-Meeri, Connie J. Mulligan

**Affiliations:** 1 Genetics and Genomics Graduate Program, University of Florida, Gainesville, Florida, United States of America; 2 University of Florida Genetics Institute, University of Florida, Gainesville, Florida, United States of America; 3 Department of Anthropology, University of Florida, Gainesville, Florida, United States of America; 4 Clinical Biochemistry Department, Faculty of Medicine and Health Sciences, Sana'a University, Sana'a, Republic of Yemen; University of Florence, Italy

## Abstract

Population migration has played an important role in human evolutionary history and in the patterning of human genetic variation. A deeper and empirically-based understanding of human migration dynamics is needed in order to interpret genetic and archaeological evidence and to accurately reconstruct the prehistoric processes that comprise human evolutionary history. Current empirical estimates of migration include either short time frames (i.e. within one generation) or partial knowledge about migration, such as proportion of migrants or distance of migration. An analysis of migration that includes both proportion of migrants and distance, and direction over multiple generations would better inform prehistoric reconstructions. To evaluate human migration, we use GPS coordinates from the place of residence of the Yemeni individuals sampled in our study, their birthplaces and their parents' and grandparents' birthplaces to calculate the proportion of migrants, as well as the distance and direction of migration events between each generation. We test for differences in these values between the generations and identify factors that influence the probability of migration. Our results show that the proportion and distance of migration between females and males is similar within generations. In contrast, the proportion and distance of migration is significantly lower in the grandparents' generation, most likely reflecting the decreasing effect of technology. Based on our results, we calculate the proportion of migration events (0.102) and mean and median distances of migration (96 km and 26 km) for the grandparent's generation to represent early times in human evolution. These estimates can serve to set parameter values of demographic models in model-based methods of prehistoric reconstruction, such as approximate Bayesian computation. Our study provides the first empirically-based estimates of human migration over multiple generations in a developing country and these estimates are intended to enable more precise reconstruction of the demographic processes that characterized human evolution.

## Introduction

Humans' facility for dispersal has played a large role in our evolutionary history, yet our understanding of how and why humans have moved throughout history is unclear. Most data on human movement come from ethnographic and archaeological studies, comparisons of birthplaces from birth certificates, and census data. While ethnographic studies offer insight into social and environmental factors that influence human movement, they generally involve seasonal or temporary movements, as in the case of migrant workers [1] or hunter- gatherers [2], [3]. In order to understand how migration has influenced our evolutionary history, it is necessary to address migration as the movement to a new location for permanent settlement. Although archaeological studies can provide information about movement over longer periods of time, they are often limited by the availability of data [4] and restricted to specific regions and time periods. Birth certificate and census data allow us to trace movement across longer periods of time as well, but studies using these data generally focus either on the proportion of migrants or the distance moved, do not usually use multi-generational families, and can typically only be studied in developed countries [5]–[10]. A deeper understanding of migration over multiple generations in a developing country offers the possibility of describing more general patterns of human migration and of identifying factors that may have influenced migration throughout human evolution.

Since human migration has had the largest effect on genetic variation over human evolution [11], a better understanding of human migration patterns would allow more accurate reconstructions of demographic processes. Comparisons of empirical genetic data to simulated genetic variation generated from models that realistically represent the demographic process under study offer the possibility of reconstructing prehistoric demographic processes [12]. Values for migration parameters estimated from human migration patterns, such as the proportion of the population that is moving, could define some model parameters in order to generate more realistic demographic scenarios. The ability to include empirically-informed values to fix or set ranges on migration parameters increases the probability of identifying the best model to explain the data.

Yemen is a developing country [13] that has a heterogeneous landscape with coastal plains on the west and south, mountain ranges in the west and desert in the north, thus providing a fertile setting in which to investigate environmental factors that may have influenced prehistoric population movements. Yemen has a patrilocal and patrilineal society with a primarily shared language and religion [14], which are social factors that could play a role in migration, as well. Migration within a population of mostly agriculturalists and pastoralists should provide more realistic values of distance and proportion of migration for prehistoric movements since the advent of agriculture. The values should also provide informative lower limits for describing the migration of prehistoric hunter-gatherers, who typically exhibit more movement than agriculturalists [4].

In this study, we use GPS coordinates from birthplaces and places of residence across four generations in Yemen to calculate the proportion, the distance and the direction of migration between each generation. We test for differences in these values between the generations, we identify factors that influence migration patterns, and we discuss possible effects of the migration patterns on genetic variation. Based on our results, we provide estimates for the proportion and distance of migration in a developing country, which can define parameter values for evolutionary models used to reconstruct prehistoric demographic processes. Our use of empirical data on population movements over four generations in Yemen provides knowledge that will allow for more accurate reconstruction of prehistoric processes of migration.

## Methods

### Ethics Statement

This study has been approved by the Western Institutional Review Board Olympia, Washington (WIRB project #20070219). Samples were collected with verbal informed consent approved by WIRB. This modified inform consent was used because a majority of the population is illiterate. Only individuals who gave consent provided both saliva samples and information for the sample collection sheet, and were entered into the database of study participants.

### Samples and Data

In 2007, saliva samples were collected throughout mainland Yemen for genetic analysis. Data were also collected from each study participant on current place of residence, place of birth, parents' place of birth and grandparents' place of birth. Since all sampled individuals were adults, their current residence was used as a proxy for the location of the next generation, i.e. their offspring, therefore providing data on residence patterns for four generations in the study. For the purposes of this study, the individuals in each generation were considered independent samples. Location names for all birthplaces (and place of residence) were translated from Arabic and GPS coordinates were obtained using Geonames.org. In instances where a town name was not identifiable in the Geonames database, but the larger district could be identified, a GPS coordinate was obtained for the centroid of the district. Samples for which town or district locations could not be determined were removed. Ultimately, the resulting dataset contained GPS coordinates for the sampled individual's place of residence and place of birth, mother's and father's places of birth, and maternal-grandmother's, maternal-grandfather's, paternal-grandmother's, and paternal-grandfather's places of birth for 351 sampled individuals (2,457 total sample locations).

### Estimation and Analysis of Migration

The occurrence of migration was determined by the difference in birthplace or residence location between generations. The current place of residence, considered a proxy for the “offspring” generation (G0) of the sampled individuals, was used to identify migration in the sampled individual's generation (G1). Thus, a migration event occurred in the sampled individual's generation (G1) if the place of residence was different from the birthplace. A migration in the parental generation (G2) occurred if the parent's offspring was born in a different location than the parent's birthplace (i.e. if the sampled individual's birthplace was different from their mother's or father's birthplace). Similarly, a migration event in the grandparental generation (G3) occurred if the parent's birthplace was different from the grandparent's birthplace. Migration events were determined for eight different groups: female sampled individuals (G1_fem_), male sampled individuals (G1_male_), mothers (G2_fem_), fathers (G2_male_), maternal-grandmothers (G3_mfem_), maternal-grandfathers (G3_mmale_), paternal-grandmothers (G3_pfem_), paternal-grandfathers (G3_pmale_). The frequency of migration events was calculated for each of the eight groups (sample sizes were 70 in G1_fem_, 281 in G1_male_, and 351 in each group in G2 and in G3. The observed frequencies were compared through goodness-of-fit tests.

The age of the sampled individuals ranged from 18 to 69, which meant that each generation group (G1, G2, G3) essentially included two generation time periods. To account for the possibility of migration events occurring over different generation time periods within each generation group, the eight groups were further divided into two age groups with a 25 year generation time between them, based on the ages of the sampled individuals (under and over 40 years old). Only 10% of the samples in any generation were in the over 40 years old sub-group, suggesting that any difference in migration event frequencies could be due instead, to the unbalanced sample size; thus no further analyses were performed with the groups partitioned by age over and under 40 years.

Migration distance was calculated from the geographic distance between birthplaces/residences in two different generations using the GPS coordinates. G1 migration distances were calculated as the geographic distance between the sampled individual's birthplace and place of residence. G2 migration distances were calculated from the parent's birthplace and the sampled individual's birthplace. Migration distances were calculated for G3 from the difference in grandparent's birthplace and parent's birthplace. The migration distances were compared between sex in each generation and between generations using Wilcoxon Rank tests and Kruskal-Wallis analyses of variance.

Different models including generation group, sex, birthplace location (latitude and longitude), and residence location (latitude and longitude) were tested in logistic regressions to see which model (and parameters) best explained migration. AIC (Akaike information criterion) were used to select the best model. Additionally, the migration events were plotted geographically and the mean direction of the migrations was calculated for each collection site (to account for sampling) using ESRI ArcMap10 [15].

## Results

**Figure 1:**
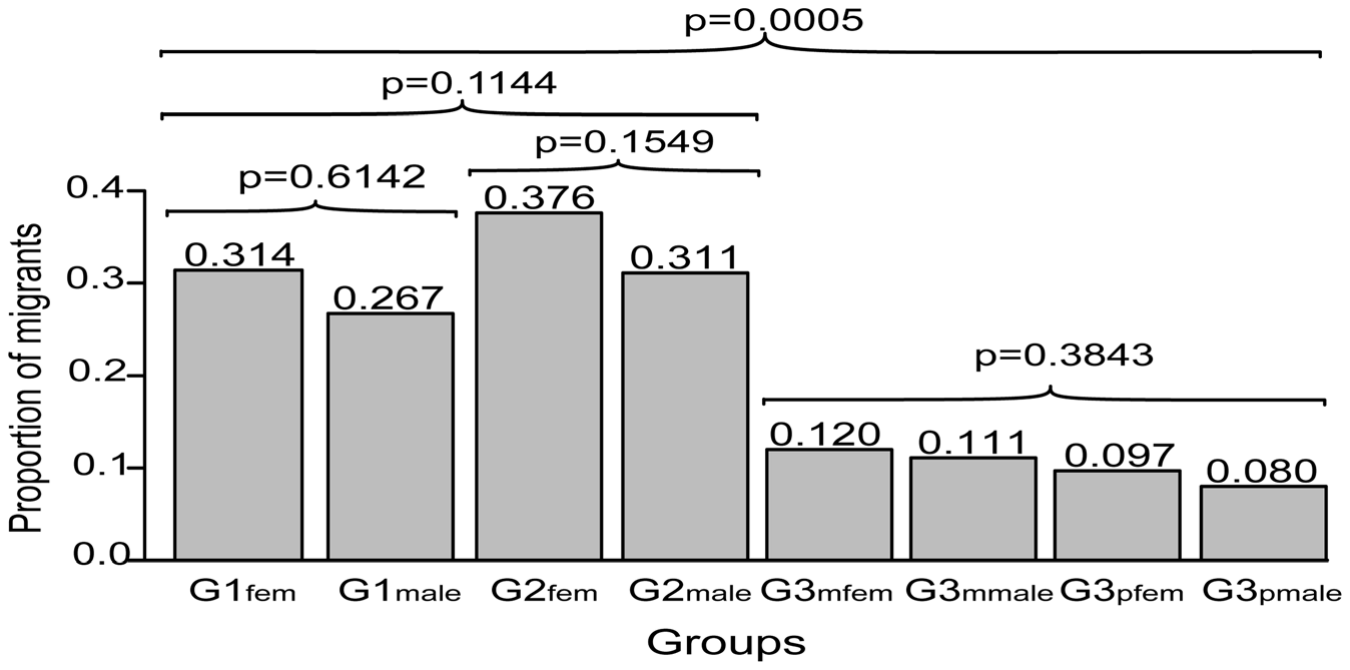
Proportion of migrants by sex for each generation group. P-values are shown for goodness-of-fit tests between groups.

The proportion of migrants was calculated from the frequency of migration events for females and males in three generations (G1_fem_ = 0.314, G1_male_ = 0.267, G2_fem_ = 0.376, G2_male_ = 0.311, G3_mfem_ = 0.120, G3_mmale_ = 0.111, G3_pfem_ = 0.097, G3_pmale_ = 0.080) (Figure 1). Within each generation, the proportion of migrants between male and female groups was not significantly different. However, more recent generations G1 and G2 had a significantly larger proportion of migrants than G3 (p = 0.0005). The proportion of migrants for each generation (males and females combined) was G1 = 0.276, G2 = 0.343, G3 = 0.102. We also calculated a multi-generation proportion of migrants for G3 to correct for back migration events by determining the number of migration events in which the grandparents' birthplace was different than the residence location. This produced a multi-generation proportion of migrants for G3 of 0.086.

**Figure 2:**
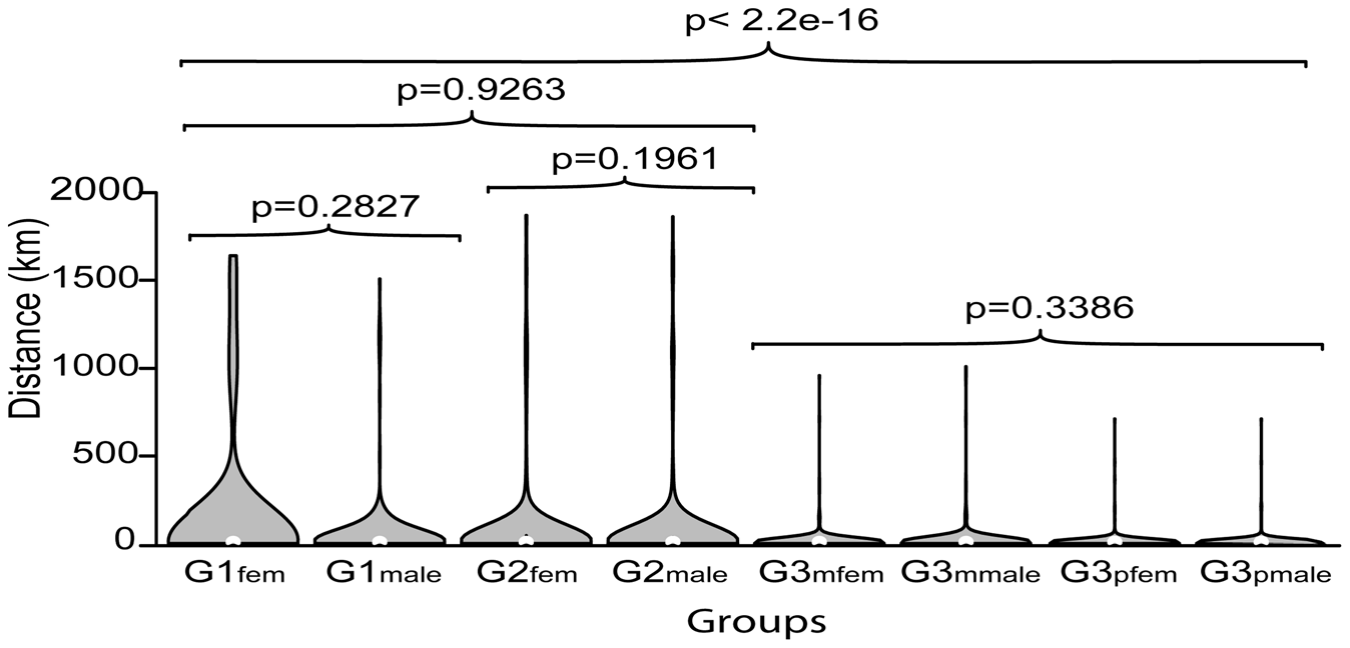
Density plots combining migration distance and frequency of the distance for each group. Wilcoxon Rank tests were performed for G1 and G2 within generation comparisons and Kruskal-Wallis tests were performed for G3 within generation comparison and between generation comparisons. P-values are shown for the respective tests.

The distance of migration was also calculated for each of the eight groups. G1 and G2 migration distances were significantly larger than G3 (p<2.2×10^−16^) (Figure 2). Density plots combining the migration distance (including non-migrants) and the frequency of these distances revealed that G1_fem_ not only had the largest migration distance, but had more migrations at longer distances (>250 km), than the other groups. However, when compared by sex within generations, female distances were not significantly different from male distances. Summary statistics on migration distances were calculated on all individuals and on only migrating individuals (Table 1).

**Table 1:** Summary statistics for migration distances.

All individuals	Mean^a^	±SD	Median^a^	Mode^a^
G1 (351^b^)	69	249	0	0
G1Female (70)	156	405	.	.
G1Male (281)	48	186	.	.
G2 (702)	72	265	.	.
G2Female (351)	73	269	.	.
G2Male (351)	72	262	.	.
G3 (1404)	10	66	.	.
G3Female (702)	9	61	.	.
G3Male (702)	10	71	.	.

Migrating individuals	Mean	±SD	Median	Mode
G1 (97)	251	424	81	103
G1Female (22)	497	601	103	103
G1Male (75)	179	328	75	103
G2 (241)	211	419	29	103
G2Female (132)	193	411	23	103
G2Male (109)	232	430	44	103
G3 (143)	96	188	26	26
G3Female (76)	82	169	24	17
G3Male (67)	111	208	28	26

Median and mode for “All individuals” was zero for all groups.

a Distances in km.

b Numbers in parentheses represent sample size.

**Figure 3:**
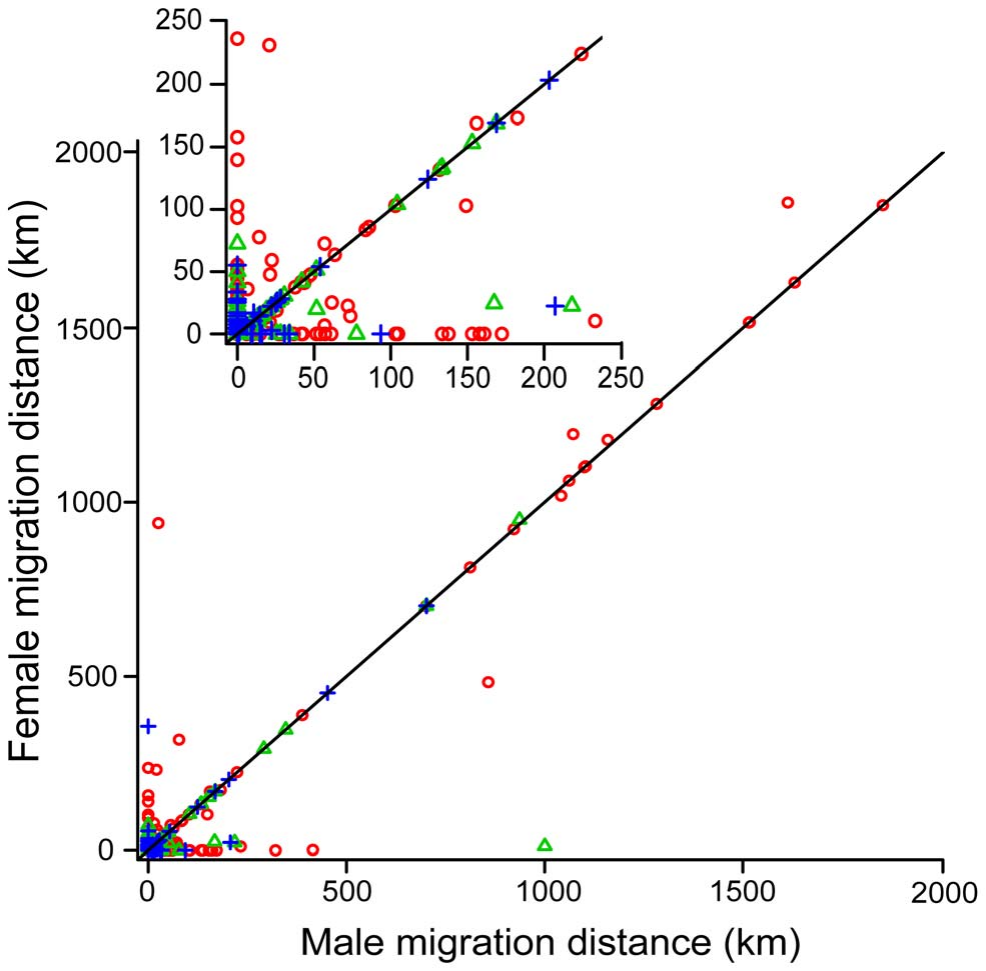
Plot of migration distances for marital pairs. G2 (red circle), G3_M_ (green triangle), G3_P_ (blue cross). The solid line shows a theoretical 1∶1 relationship, where females and males have the same dispersal distance. The inner box shows a close-up of the relationship for distances less than 250 km.

Correlation analyses were performed on marital pairs in G2 and G3 to determine whether marital pairs were moving together. A low correlation coefficient (<0.1) would suggest the marital pair migrations were completely independent from each other and a high correlation coefficient (>0.9) would suggest that the marital pairs were moving together and should be treated as one group (instead of female and male groups). G2 had a significant (p = 2.2×10^−16^) Spearman's rho correlation coefficient of 0.589. Maternal grandparents (G3_M_) had a rho coefficient of 0.782 (p = 2.2×10^−16^) and paternal grandparents (G3_P_) had a rho coefficient of 0.623 (p = 2.2×10^−16^). These results showed there was a moderate and significant correlation between all marital pairs. These coefficients suggest that a portion of the marital pairs are moving together, but the correlations are not high enough (>0.9) to consider the marital pairs as a single group. Female and male marital pair distances were plotted and showed that correlated migrations were of the same distance, which is consistent with marital pairs moving to the same place (Figure 3). Out of the 121 migration events in G3, 56% were of marital pairs moving together. These results suggest that many of the individuals may be moving due to post-marital residence dynamics (i.e. husbands and wives moving together).

Logistic regression models, including different combinations of generation, sex, birthplace coordinates and residence location coordinates, were performed to explain presence or absence of migration. The model with the lowest AIC included generation, sex, birth latitude and longitude and residence latitude (Table 2). This best model demonstrated, that relative to G1_fem_ (as the baseline group), the probability of migration decreased in G3, decreased in males (consistent with females moving with their husbands' families) and decreased with a more easterly birthplace. In contrast, the probability of migration increased in G2 and increased with more northern birthplaces and places of residence. However, of these factors, only G3 had a coefficient above one, suggesting that G3 contributes the most to the probability of migration, and specifically, belonging to the G3 generation decreases the probability of migration.

**Table 2:** Best model to explain probability of migration.

Factor	Coefficient
Intercept	−1.924
Generation:G2	0.256
Generation:G3	−1.244
Sex:Male	−0.240
Birth Latitude	0.221
Birth Longitude	−0.121
Residence Latitude	0.225

p<0.04 for all factors. The probability of migration decreases in G3, decreases in males, and decreases with a more eastern birthplace, in comparison to G1_fem_. The probability of migration increases in G2 and increases with more northern birthplaces and places of residence.

**Figure 4:**
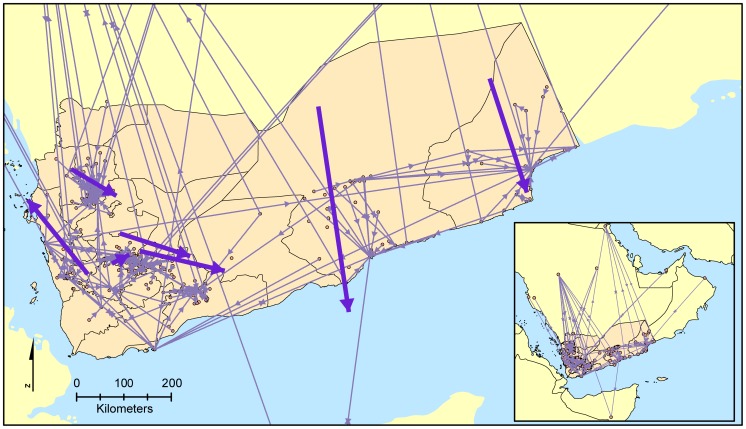
Migration direction vectors and mean migration direction by collection site over all three generations. Small arrows represent migration vectors and large arrows represent mean migration direction.

Although birthplace latitude, birth place longitude and residence location latitude had small coefficients, their statistically significant contribution to the migration probability suggests that there could be factors “pushing” individuals away from a place (leave one's birthplace) or “pulling” individuals to a place (move to a new place) [16]. The birthplace and residence coordinates were used to plot the directionality of migration to assess whether or not there was a pattern in directionality that could explain the “pushing” and “pulling” effects (Figure 4). The mean migration direction was calculated from these migration vectors for each sample collection site (to account for the effect of sampling). While the mean migration directions seem to have a southbound tendency, the circular variance (which describes the variation associated with the directional mean, where values close to 0 represent a similar direction for all migration vectors and values close to 1 correspond to vectors in all compass directions) was moderate to high for all collection sites, ranging from 0.675 to 0.867 (Table 3), suggesting movement in all directions.

**Table 3:** Estimates for the direction of migration in each collection site across all three generation groups.

Collection site	Mean directional angle^a^	Mean distance^b^	Circular variance^c^
Abyan	103.19	1.659	0.676
Al Bayda	107.87	1.405	0.806
Al Hudaydah	321.06	1.814	0.867
Al Mahra	161.96	2.282	0.833
Amran	120.51	0.995	0.704
Dhamar	62.78	0.370	0.758
Hadramout	171.57	3.953	0.675

a Mean directional angle is measured clockwise from due North.

b Mean distance is measured in decimal degrees.

c Circular variance describes the variation associated with the directional mean, where values close to 0 represent a similar direction for all migration vectors and values close to 1 correspond to vectors in all compass directions.

The mean migration directions were further calculated by collection site for each generation group (Figure S1 and Table S1). Within generation groups G2 and G3, female and male migration directions were similar in many collection sites, supporting the idea that marital pairs moved together. The mean migration lengths were generally larger for G1 and G2 than for G3, reflecting the decreased migration distance in G3. For each collection site, the mean migration directions varied greatly between generation groups, suggesting a level of stochasticity to the migration directions. When the mean migration directions were spatially compared to geographic features (i.e. elevation, land use/land cover, and watershed), no pattern arose (data not shown), further supporting stochasticity in the directionality of migrations.

## Discussion

Our study helps elucidate human migration patterns using empirical population movement data across multiple generations in Yemen. Our results show that the proportion and distance of migration increased in recent generations. While movement in the recent generations may reflect social and political changes that have occurred in the last 50 years [17], the reduced movement in the oldest generation most likely reflects a lack of technology and associated mobility [16], suggesting that this generation may be most representative of prehistoric movements. The correlated distance and directionality of migrations within marital pairs illustrate the prevalence of post-marital residence dynamics. The significance of birthplace and residence locations in the probability of migration, but lack of pattern in the direction of migration, suggest a degree of stochasticity in terms of human movements. These cultural factors affecting modern movement have most likely played important roles in prehistoric migrations as well, suggesting that the migration patterns and estimates described in our results provide information to make more accurate prehistoric inferences.

### Patrilocality and Genetic Signals

Moderate correlation coefficients for G2 and G3 marital pairs and the plot of migration distances in marital pairs suggests that pairs are moving together and the correlation seems to strengthen with increasing distance (Figure 3). Our best fit model, which shows that females are more likely to move than males when accounting for other contributing variables, suggests that patrilocality (females moving to their husbands' family) may be driving the movement. This is supported by ethnographic accounts that ∼90% of the Yemeni population is patrilocal [18]. However, the coefficient of the effect that being male has on the probability of migration is low (−0.240) and within each generation the migration distance is not significantly different between females and males. This suggests that males are only slightly less likely to migrate than females and that males are travelling similar distances compared to females. In a perfect patrilocal post-marital residence dynamic, males move short distances and stay close to their family, while females move longer distances to be near their husbands' family. The similar migration distances between females and males suggest there is not strict patrilocality in Yemen and that other factors are influencing male movement. This interpretation is supported by ethnographic data showing that males may occasionally migrate large distances from their birthplace for socioeconomic or political reasons [14], [18]. Our data show that male migration has occurred more often in the last 50 years (as shown by the increase in dispersal in G1 and G2 relative to G3).

The similar migration distances between females and males, and consequent imperfect patrilocality may be the principal contributor to the lack of association observed between geographic and genetic distance in male lineages (i.e. Y chromosome) in Yemen [19]. Females moving with their husbands may also explain why shared mitochondrial DNA (mtDNA) haplotypes have been found between east and west Yemen, over 750 km apart [20].

### Patterns of Migration

Logistic regressions were used to test the effect of birthplace and residence locations on the probability of migration in order to assess whether there were factors “pushing” or “pulling”, respectively, individuals to a new location. Birthplace latitude and longitude and residence latitude were significant parameters in explaining the probability of migration. Given this result, birthplace and residence coordinates were used to plot migration directions and determine whether a pattern could be observed that could account for the effects of birthplace and residence locations. Mean migration directions were calculated by collection site (to account for sampling bias) to summarize the overall migration direction patterns (Figures 4 and S1, and Table 3). Although the mean migration directions had a southbound trend, the circular variances were large, suggesting overall dispersal in multiple directions (Figure 4 and Table 3). Additionally, mean migration directions calculated by collection site for each generation showed that the collection sites had different mean directions between generations, further supporting migration in multiple directions (Figure S1 and Table S1). We also spatially compared the migration directions with different geographic features (i.e. elevation, land use/land cover, and watershed) to identify environmental factors that may influence migration direction. We found no pattern associated with the migration directions and the geographic features (data not shown). These results suggest that while there may be factors “pushing” and “pulling” individuals to move, the overall direction of migration has little or no pattern. These results contrast with island migration patterns (e.g., Polynesia) where migration direction has a pattern from larger islands to smaller islands [21]–[23]. Given that continental migrations are less limited by the carrying capacity of new colonization sites than islands, our results are not surprising.

While island migrations have been well described by ethnographic and archaeological data [21], [23], continental migration patterns have been primarily addressed through genetic data. Genetic evidence has suggested that overall continental migrations have a linear pattern, such that increasing distance from Africa is correlated with decreasing genetic diversity [24], [25]. Our data suggest that the smaller scale migrations (Figures 4 and S1) that led to this continental pattern may have been less directed. Our results are consistent with the idea that smaller migrations, which consider the movement of individuals, tend to be more random, while larger scale movements focused on populations have more directionality associated with them [4], [26].

### Empirical Estimates of Migration

Comparisons of proportion of migrants and migration distances across four generations showed that migration was significantly lower around fifty years ago (G3). Furthermore, the best fit model to explain the probability of migration shows that G3 has not only the biggest effect, but a negative effect on the probability of migration (i.e. belonging to G3 decreases the probability of a migration event). Spatial patterns of migration in G3 (Figure S1c) show, that although there are some long migration distances, on average, the distances are short. Yemen's less-developed state and poor transportation infrastructure [17] combined with the significantly reduced migration in G3, suggests that our data from the G3 generation can provide empirically-based estimates of migration frequency and distance that are reflective of prehistoric movements.

We calculated the mean and median migration distances for G3 (Table 1). The mean migration distance for all individuals (i.e. including both individuals who migrated and those who did not) was 10 km. The mean and median distances for migrating individuals only were 96 km and 26 km, respectively. The shorter migration distance values (10 km and 26 km) are within the range of previously reported average migration distances [27]–[29]. These shorter migration distances potentially demarcate the distances within which post-marital residence patterns (patrilocality in the case of Yemen) have a distinguishable effect on genetic structure [19], [29]. In contrast, at distances beyond these values, isolation by distance is probably more predominant, and sex-biased migration is less detectable.

Since most populations before the advent of agriculture (∼10 kya) were hunter-gatherers, we wished to identify whether our results provided estimates that may be informative in reconstructing prehistoric processes throughout these different periods in human history. Our shorter migration distance values (10 km and 26 km) are within the range of 10–30 km that Ammerman and Cavalli-Sforza [27] believe is plausible for migration distance in agriculturalist societies. Furthermore, dividing 26 km by a generation time of 25 years results in a migration speed of 1.04 km/year. This value is comparable to the 1 km/year migration speed for the Neolithic transition estimated from archeological data [4], [30]. These similarities suggest that the shorter distance values, particularly the median distance, are representative of migration distances of agriculturalist groups.

Hunter-gatherers generally migrate more and longer distances than agriculturalists. Therefore, our mean migration distance estimated using only migrating individuals offers a potentially informative migration value for the more mobile hunter-gatherer populations. Specifically, a migration speed (3.84 km/year) calculated from the mean value for only migrating individuals (96 km) falls within the broad range of hunter-gatherer migration speeds based on archeological evidence. Fort et al [31] estimated the speed of the hunter-gatherers' recolonization of northern Europe after the last glacial maxima between 0.7 and 1.4 km/year. Hamilton and Buchanan [32] estimated a speed of 5–8 km/year for the colonization of North America, while Hazelwood and Steele [4] obtained estimates of 6–10 km/year. Because our value is intermediate to the values of these region-specific studies, it provides a distance that may be more generally applicable to migration processes, particularly *de novo* colonization migration distances by hunter-gatherers. This can be seen when we compare our migration speed estimate with Macaulay et al's [33] inferred migration speed for the colonization of Southeast Asia. Based on founder time estimates from Eurasian and Australasian mtDNAs and the distance between India and Australasia, Macaulay et al infer a migration speed of 4 km/year. Our empirical estimate of 3.84 km/year suggests that their proposed migration process is in fact plausible.

While migration distance has been estimated through different approaches, few studies have estimated the proportion of migrants [5], [7], [29], [34]. We calculated the proportion of migrants for G3 to be 0.102 (or 0.086 when adjusting for back migration in the four generations). These values are smaller than the 0.4 proportion of migrants that can be calculated from Wood et al's [34] dataset on migration between parishes in Papua New Guinea or the 0.366 estimate obtained from the calculation of individuals that were not born in the same parishes as their parents in La Cabrera, Spain [7]. These differences from our estimates seem reasonable as Wood et al's estimates are from a more recent population (and are closer to our G1 and G2 estimates) and Boatinni et al's estimates are from a more developed country. Our estimates are somewhat larger than the 0.032 proportion of migrants into the island of Pingelap in Micronesia presented by Morton et al [5]. However, our adjusted proportion of migrants (0.087) is closer to Morton et al's value. We also calculated the maximum and average number of individuals moving between a pair of locations, for a proportion of migrants of 0.0036 and 0.0011, respectively. These lower values are consistent with findings by Deshpande et al [35], where the genetic estimates of proportion of migrants (i.e. migration rates) for a world-wide colonization model are less than 0.01. Our values are similar to findings by Miró-Herrans and Mulligan [11], where the most probable proportion of migrants exchanged between African and non-Africans populations was 0.001 and are similar to the migration rate for non-African populations (1.5×10^−3^) obtained by Cox et al [36]. The similarity of our estimates with those from other migration studies suggests that our values can be used in different scenarios to generate testable models for prehistoric reconstruction.

### Application of Migration Estimates in Prehistoric Demographic Modeling

Model-based approaches for inferring prehistoric processes from genetic variation are becoming increasingly popular [37]. These approaches, such as approximate Bayesian computation [12], require the generation of explicit demographic models to compare to empirical data. Including specific values for known parameters and informative ranges of values for unknown parameters increases the probability of identifying the best model to explain the data. The results from our study provide estimates that can be used to fix or set ranges on parameters related to migration, such as gene flow or founding population size, so that other parameters of interest can be addressed in greater depth, e.g., time of a demographic event. For example, the maximum and average proportion of individuals moving between a pair of locations (0.0036 and 0.0011) can be used to define gene flow (or migration rates) between populations stretching from southern Asia to northern Africa to create simulated DNA for models that address the back-migration into Africa. The larger migration values (0.102 or 0.086) can be used to define the founding population sizes for each new population out-of-Africa and back-to-Africa. Defining these parameters would allow for an in-depth exploration of the timing of the back-migration.

Additionally, our results provide estimates to generate more geographically explicit models. Our mean and median migration distances (96 km and 26 km) provide estimates for the distance between populations, particularly for large scale movements, such as the back-migration from southern Asia. The migration distance between each population would define the number of populations to be simulated for the region under study. For example, a distance of 100 km between each population would require ∼70 populations between southern Asia and northern Africa (approx. 7,000 km). Understanding the possible distances involved in large scale movements also helps us determine how rapidly a migration could have occurred and how levels of gene flow may have been affected between the populations.

The lack of migration directionality in our results suggests that explicitly including stochasticity or multidirectionality when describing the movement between populations might more accurately reflect the large-scale migration process. For example, the back-migration to Africa probably included movement through established populations, where the migrants settled in some of the established populations, but not in others. Therefore, a lattice stepping-stone migration model, that includes some randomness in terms of when a migration occurs and between which populations, might better reflect this migration process.

Our results show there is over a 58% correlation between female and male movement in marital pairs, in which more pairs move together with increasing distance. Additionally, we show that 56% of migration events in G3 were by marital pairs. This means that at least 50% of the migrants have a 1∶1 female to male ratio. Even if the remaining 50% of migrants are only female or male, the ratio is at most 3∶1. These results argue for, at most, a 3∶1 ratio (for either sex) of sex-biased migration for migrations at short distances, where post-marital residence has a larger effect on population structuring [19], [29]. Alternatively, for longer migrations, such as the migration from southern Asia to northern Africa, our results suggest that a female to male ratio closer to 1∶1 more accurately models demographically balanced populations that would have been reproductively self-sustaining.

## Conclusions

In this study, we analyzed empirical data on migration patterns over four generations of human populations in Yemen in order to gain insight into the factors that influence migration, and specifically may have affected prehistoric movements throughout human evolution. Our approach to trace migration over generations has enabled the study of migration patterns throughout a developing country that would otherwise have been unfeasible. We provide empirical estimates for migration-related parameters that can be used to generate demographic models in model-based methods of prehistoric reconstruction. Our empirical estimates of generation G3 provide values for proportion of migrants, with values ranging from 0.102 or 0.086 proportion of overall migration, to 0.0036 or 0.0011 proportion of migrants between two specific populations. We also provide migration distances (96 km and 26 km, mean and median, respectively) that can be used to define the distance between populations and therefore the number of populations for the area under study. Using our approach, populations employing other modes of subsistence, such as hunter-gatherers, may be studied to further improve our knowledge on human migration.

The findings from this study shed light on human migration patterns and enable more accurate reconstruction of the demographic processes that characterized human evolution. Improved models of human demographic changes and the associated genetic variation can provide a powerful tool to test for selective pressures, as well as to model the evolutionary history of co-evolving organisms. In this way, reconstruction of human demography and evolution may further provide insight into the movement and evolution of human pathogens and other co-evolving organisms.

## Supporting Information

Figure S1 **Migration direction vectors and mean migration direction for each collection site by generation group.** a)G1. b)G2. c)G3. Females: purple, Males: green. Small arrows: migration vectors. Large arrows: mean migration direction.
(TIF)

Table S1 **Directional means estimates for each group by collection site.**
(DOCX)
