# Supplementary figures and images for: Human Migration Patterns in Yemen and Implications for Reconstructing Prehistoric Population Movements
Source: PLoS One. 2014 Apr 23;9(4):e95712. doi: 10.1371/journal.pone.0095712 (PMC3997431; doi:10.1371/journal.pone.0095712)

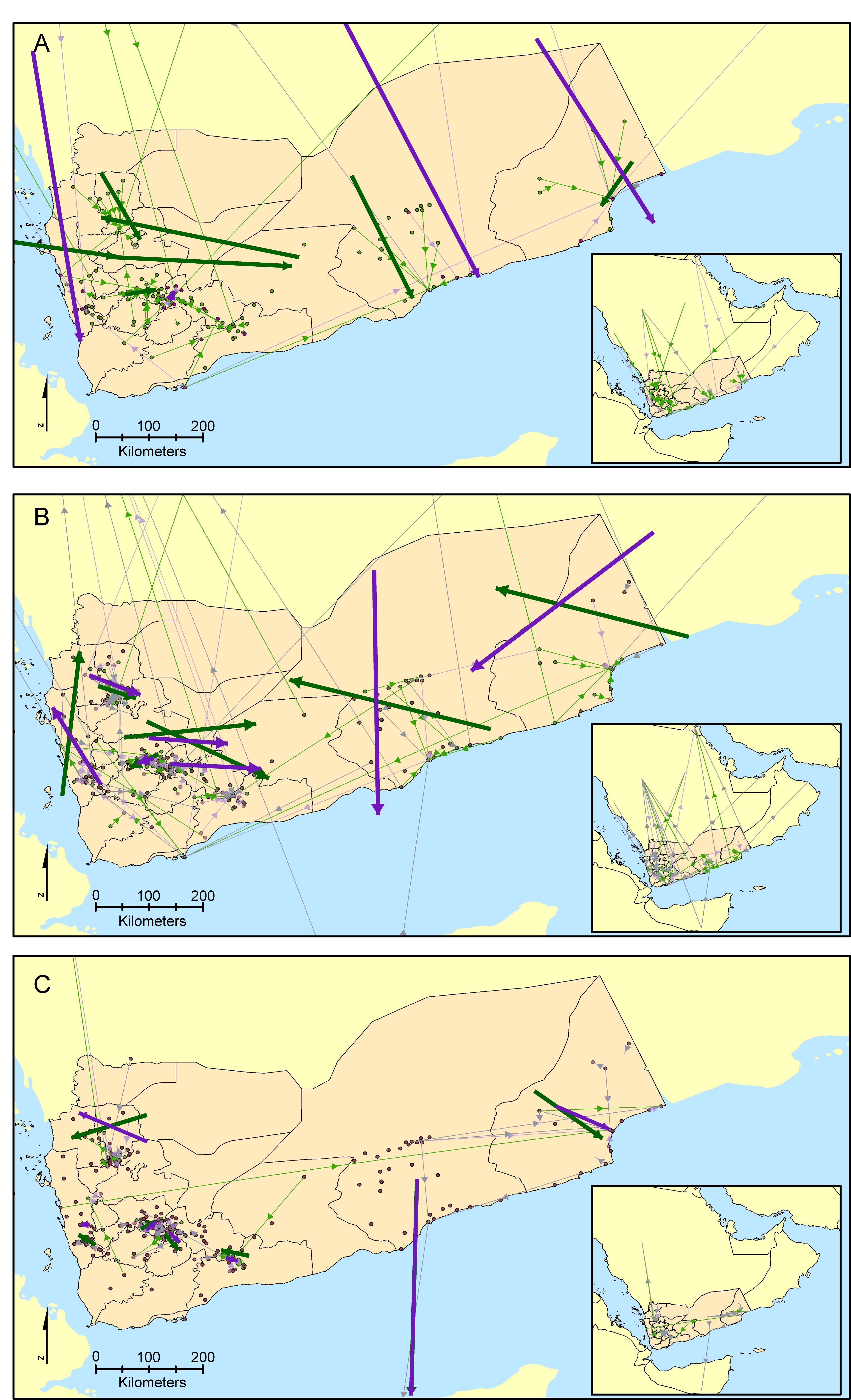

Supplement: Figure S1 — Migration direction vectors and mean migration direction for each collection site by generation group. a)G1. b)G2. c)G3. Females: purple, Males: green. Small arrows: migration vectors. Large arrows: mean migration direction. (TIF) [file pone.0095712.s001.tif]
