# Supplementary material for: Human Migration Patterns in Yemen and Implications for Reconstructing Prehistoric Population Movements
Source: PLoS One. 2014 Apr 23;9(4):e95712. doi: 10.1371/journal.pone.0095712 (PMC3997431; doi:10.1371/journal.pone.0095712)
Supplement: Table S1 — Directional means estimates for each group by collection site. (DOCX) [file pone.0095712.s002.docx]

Table S1. Directional means estimates for each group by collection site.

| **Group** | **Generation** | **Collection site** | **Mean directional angle^a^** | **Circular variance** | **Mean distance^b^** |
| --- | --- | --- | --- | --- | --- |
| G1female | G1 | Al Bayda | 20.676 | 0.045 | 0.174 |
| G1female | G1 | Al Hudaydah | 171.328 | 0.471 | 5.307 |
| G1female | G1 | Al Mahra | 149.404 | 0.714 | 3.869 |
| G1female | G1 | Hadramout | 153.945 | 0.023 | 9.225 |
| G1male | G1 | Abyan | 93.105 | 0.513 | 3.115 |
| G1male | G1 | Al Bayda | 282.164 | 0.914 | 3.400 |
| G1male | G1 | Al Hudaydah | 98.593 | 0.603 | 1.960 |
| G1male | G1 | Al Mahra | 213.058 | 0.494 | 0.956 |
| G1male | G1 | Amran | 151.418 | 0.640 | 1.382 |
| G1male | G1 | Dhamar | 79.936 | 0.522 | 0.515 |
| G1male | G1 | Hadramout | 155.123 | 0.430 | 2.441 |
| G2female | G2 | Abyan | 93.264 | 0.675 | 1.525 |
| G2female | G2 | Al Bayda | 94.257 | 0.773 | 1.335 |
| G2female | G2 | Al Hudaydah | 329.910 | 0.795 | 1.620 |
| G2female | G2 | Al Mahra | 230.602 | 0.853 | 3.961 |
| G2female | G2 | Amran | 112.443 | 0.645 | 0.931 |
| G2female | G2 | Dhamar | 251.241 | 0.781 | 0.290 |
| G2female | G2 | Hadramout | 179.180 | 0.895 | 4.442 |
| G2male | G2 | Abyan | 116.708 | 0.539 | 2.298 |
| G2male | G2 | Al Bayda | 83.530 | 0.827 | 2.226 |
| G2male | G2 | Al Hudaydah | 6.450 | 0.765 | 2.635 |
| G2male | G2 | Al Mahra | 285.254 | 0.926 | 3.355 |
| G2male | G2 | Amran | 109.280 | 0.400 | 0.675 |
| G2male | G2 | Dhamar | 243.264 | 0.895 | 0.540 |
| G2male | G2 | Hadramout | 284.876 | 0.952 | 3.493 |
| G3female | G3 | Abyan | 106.306 | 0.522 | 0.200 |
| G3female | G3 | Al Bayda | 135.958 | 0.631 | 0.255 |
| G3female | G3 | Al Hudaydah | 264.634 | 0.643 | 0.148 |
| G3female | G3 | Al Mahra | 114.916 | 0.698 | 0.973 |
| G3female | G3 | Amran | 294.408 | 0.765 | 1.258 |
| G3female | G3 | Dhamar | 48.563 | 0.530 | 0.232 |
| G3female | G3 | Hadramout | 181.400 | 0.006 | 3.917 |
| G3male | G3 | Abyan | 281.966 | 0.846 | 0.479 |
| G3male | G3 | Al Bayda | 141.926 | 0.633 | 0.371 |
| G3male | G3 | Al Hudaydah | 303.292 | 0.583 | 0.339 |
| G3male | G3 | Al Mahra | 126.886 | 0.757 | 1.427 |
| G3male | G3 | Amran | 252.032 | 0.689 | 1.334 |
| G3male | G3 | Dhamar | 61.057 | 0.557 | 0.292 |
| G3male | G3 | Hadramout | 181.400 | 0.006 | 3.917 |

^a^Mean directional angle is measured clockwise from due North. ^b^Mean distance is measured in decimal degrees.
